# Senescence associated macrophages and “macroph-aging”: are they pieces of the same puzzle?

**DOI:** 10.18632/aging.101133

**Published:** 2016-12-07

**Authors:** Francesco Prattichizzo, Massimiliano Bonafè, Fabiola Olivieri, Claudio Franceschi

**Affiliations:** Department of Clinical and Molecular Sciences (DISCLIMO), Università Politecnica delle Marche, 60020 Ancona, Italy

**Keywords:** aging, macrophages, age-related diseases, longevity, senescence

Human aging is characterized by a failure to maintain tissue homeostasis. Mounting evidence suggests that the accumulation of senescent cells (SCs) is a causal agent in this process, highlighting the relationship between increased SC burden, loss of homeostasis, and heightened risk of developing age-related diseases (ARDs) [1]. Exploring the mechanisms that link SCs and tissue homeostasis is essential to devise effective strategies to slow down the age-related decline and degeneration of human tissues. Cellular senescence is known to be induced by the activation of compensatory/antagonistic responses to the age-related cellular damage caused by a number of stressors, like radiations and oncogenes activation. SCs activate a pro-inflammatory program characterized by release of a number of soluble factors—hence the name senescence-associated secretory phenotype (SASP)—whose main goal is to achieve the immune-mediated clearance of SCs [2]. Intriguingly, the SASP can transmit senescence to bystander cells, resulting in an increased number of SCs and perpetuation of the chronic, low-grade, age-related inflammatory state that some years ago we have defined “inflammaging” [3]. Different phenomena seem to be involved in the age-related increase in the SC burden associated with inflammaging, including faster spread of senescence in older compared with younger organisms and less efficient SC clearance during aging. The two mechanisms are not mutually exclusive; indeed, the most likely hypothesis is that they coexist, although convincing data have yet to be provided.

Now, Hall and co-workers have demonstrated that SCs can spread senescence to immune cells [4]. By implanting into the peritoneal cavity of SCID mice senescent fibroblasts capable of releasing a small bioluminescent protein, the authors showed that acquired immunity is not mandatory for SC clearance. Inoculation of alginate bead-embedded SCs into p16LUC mice (which have normal immunity) indicated that SCs attracted immune cells, including macrophages. Since alginate coating allows only effusion of the soluble molecules released from SCs (i.e. SASP factors), these soluble components can attract immune cells. Surprisingly, this secretome induced acquisition by young mouse macrophages of a senescence-like phenotype characterized by an increased expression of tumor suppressor p16(Ink4a) and activity of β-galactosidase (β-gal) at pH 6.0 (β-galpH6), two markers of senescence that have proved highly reliable *in vitro* and *in vivo* studies. Moreover, the p16+/β-gal+ cell subset was found to be the population identified as SCs in chronologically aged p16LUC mice. A considerable fraction of these cells were cleared by liposomal clodronate, which kills cells with phagocytic activity. These cells were defined senescence associated macrophages (SAMs) [4]. The possibility that clodronate treatment affected SC types other than macrophages cannot be totally excluded, since fibroblasts and endothelial cells also have a certain phagocytic activity and become senescent both *in vivo* and during *in vitro* replicative senescence [2]. However, when Hall and co-authors analyzed the adipose tissue of old mice for SCs before and after clodronate treatment, they found a large reduction in β-gal+ cells. In addition, most β-gal+ cells expressed F4/80, a well-known and widely used marker of murine macrophage populations, thus reinforcing the notion that most SCs accumulating in fat during aging are a macrophage subset, rather than pre-adipocytes [4].

Altogether, these data demonstrate for the first time that the senescence bystander effect can be extended to immune cells, i.e. macrophages, and that their number increases in tissues of aged mice. These findings strongly support the hypothesis that macrophages play a key role in aging processes.

We have described a similar hypothesis in relation to the conceptualization of aging as the result of the chronic stress that fuels inflammaging [3]. In its framework, we stressed that macrophages are key cells in the induction and maintenance of inflammaging, and coined the term “macroph-aging” to describe the effect of the chronic macrophage activation that characterizes aging [3]. Inflammaging and macroph-aging occur in association with immunosenescence, whose hallmark is a reduced efficiency of immunity cells to cope with stressors during aging [3].

The demonstration, by Hall and colleagues, that senescence spreads to macrophages and that SAMs accumulate in old mice broadens the horizon. Now the main outstanding questions are: 1- are senescent macrophages proinflammatory cells sustaining inflammaging? 2- Do senescent macrophages retain the ability to efficiently clear the other SCs? 3- Do senescent macrophages contribute to immuno-senescence?

Answering these questions is a prerequisite for testing the hypothesis that clearance of senescent macrophages can prolong lifespan/healthspan in animal models. So far, genetic engineering and pharmacological approaches aimed to remove SCs from the organism have shown variable success in restraining inflammation in different organs, including, kidney and fat, thus increasing mice lifespan and/or healthspan. ABT263, a senolytic agent targeting both antiapoptotic proteins, BCL-xL and BCL-2, has recently been shown to induce apoptosis in SCs, rejuvenating aged bone marrow hematopoietic stem cells [5]. Interestingly clodronate treatment, which kills phagocytic cells, has been reported to induce a drastic reduction of age-associated inflammatory responses to systemic immunostimulation in aged mice [6].

Recent data on mice demonstrating that foamy macrophages with senescence markers accumulate in the sub-endothelial space, driving atherosclerotic process by increasing expression of inflammatory cytokines, chemokines, and metalloproteinases, strongly support the involvement of macroph-aging in the development of ARDs [7].

If the intriguing hypothesis that the accumulation of senescent macrophages contributes to accelerate the aging processes in humans is demonstrated, work on senolytics will need to be integrated by research into senescent macrophage-lytics.
